# Ethinyl estradiol and levonorgestrel coadministration induces hypophagia and increases energy expenditure in female rats

**DOI:** 10.1210/endocr/bqag064

**Published:** 2026-06-01

**Authors:** Jesse M Lacasse, Ahmad Mohammad, Kristen R Montgomery, Sarah Bellaflor, Rebecca E K MacPherson, Liisa A M Galea, Cheryl M McCormick

**Affiliations:** Department of Psychology, Brock University, St. Catharines, ON, Canada L2S 3A1; Campbell Family Mental Health Research Institute, Centre for Addiction and Mental Health, Toronto, ON, Canada M5T 1R8; Department of Health Science, Brock University, St. Catharines, ON, Canada L2S 3A1; Campbell Family Mental Health Research Institute, Centre for Addiction and Mental Health, Toronto, ON, Canada M5T 1R8; Department of Health Science, Brock University, St. Catharines, ON, Canada L2S 3A1; Department of Health Science, Brock University, St. Catharines, ON, Canada L2S 3A1; Centre for Neuroscience, Brock University, St. Catharines, ON, Canada L2S 3A1; Campbell Family Mental Health Research Institute, Centre for Addiction and Mental Health, Toronto, ON, Canada M5T 1R8; Department of Psychiatry, University of Toronto, Toronto, ON, Canada M5T 1R8; Department of Psychology, Brock University, St. Catharines, ON, Canada L2S 3A1; Centre for Neuroscience, Brock University, St. Catharines, ON, Canada L2S 3A1

**Keywords:** hormonal contraceptives, energy expenditure, body composition, development, adolescence, progestin

## Abstract

Oral contraceptives containing ethinyl estradiol (EE) with levonorgestrel (LNG) are common in hormonal contraceptives. EE+LNG suppresses weight gain in rodents, but the mechanisms for this remain unclear. Further, metabolic demands in adolescence are increased relative to adulthood. We tested the metabolic effect of a low dose of EE + LNG in adolescents and adult female Long-Evans rats. Rats were given EE (10 µg/kg) + LNG (20 µg/kg) or vehicle subcutaneous injections daily for 16 days. Body mass, food intake, whole-body oxygen consumption, respiratory exchange ratio, and energy expenditure were measured in Promethion phenotyping cages. Dual-energy X-ray absorptiometry (DXA), glucose tolerance, serum metabolic hormone levels, depot-specific adipose and reproductive tissues were collected.

EE+LNG limited body-mass gain in both age groups by combining hypophagia with a marked elevation in total energy expenditure and no change in locomotion, implicating potential thermogenic pathways. Dual-energy X-ray absorptiometry scans showed curtailed fat accrual and the preservation of lean mass in EE + LNG-treated rats. White-adipose depots were smaller in all hormone-treated rats, whereas brown adipose tissue mass was reduced only in adults. EE+LNG-treated adults had lower leptin and C-peptide levels with enhanced glucose clearance, whereas adolescents had reductions in GLP-1, glucagon, and PYY levels without altered glucose clearance. Thus low-dose EE + LNG reduced caloric intake, increased energy expenditure, and limited white-adipose deposition irrespective of age. Age-specific effects on metabolic hormones and adipose depot UCP1 content were noted. Together, these results delineate an age-sensitive metabolic and endocrine profile of EE + LNG.

Hormonal contraceptives (HCs) are among the most widely used medications, but their systemic metabolic profile is only partly defined. Despite the common perception that HCs induce weight gain, the best available evidence indicates no clinically meaningful, population-level change in body weight for most users (1-3). When weight shifts occur, they are typically small, often including a modest early decrease that later returns toward baseline (3, 4). In rodents, repeated administration of the combination of ethinyl estradiol (EE) and levonorgestrel (LNG), the most common HC formulation prescribed in North America (5, 6), reliably suppresses body-mass gain (7-16). A growing set of preclinical rodent studies have begun to quantify metabolic endpoints in response to HC (12, 17, 18); however, the available data are inconsistent. Although it is well established that steroid hormones such as estrogens, progestogens, and androgens play key roles in regulating body mass and energy balance (19, 20), the mechanisms underlying the combination of EE with LNG (EE+LNG)-induced weight suppression in rodents remain largely uncharacterized.

Endogenous gonadal hormones play a multifaceted role in the regulation of body mass and energy balance. These hormones influence several physiological processes relevant to regulating body mass, including energy metabolism (21, 22), energy expenditure, and locomotor activity (23, 24). They also modulate feeding-related behaviors, affecting not only the quantity of food consumed (25, 26), but also appetite and hedonic responses to palatable foods (27-29) and fluid regulation (30). Taken together, these findings suggest that fluctuations in endogenous gonadal hormone levels may substantially alter energy homeostasis and feeding behavior, thereby affecting weight regulation.

Previous research suggests that synthetic hormones such as EE and LNG may disrupt energy balance (7, 14), but it is unclear whether the observed reduction in weight gain is due to decreased caloric intake, increased energy expenditure, or both. We investigated these possibilities by administering EE and LNG in combination to female rats. An additional consideration was that most HC studies are conducted in adult animals, even though metabolic demands differ markedly between adolescence and adulthood (31, 32). Because combined HCs suppress the hypothalamic-pituitary-gonadal (HPG) axis, exposure during adolescence could plausibly alter somatic growth. Indeed, recent work shows that EE + LNG blunts weight gain in adolescent rats (13), underscoring the need to evaluate their effects during development. We therefore included an adolescent cohort to test whether younger rats are more susceptible to the weight-related actions of EE + LNG, because relatively little is known about the metabolic effects of HC exposure during adolescence, a period of ongoing physiological maturation that may confer heightened sensitivity to synthetic hormonal perturbation. We hypothesized that EE and LNG treatment would reduce body mass through both reduced caloric intake and increased energy expenditure, which would differ between age groups.

## Materials and methods

### Test subjects

Sixty-four female Long-Evans rats were obtained from Charles River Laboratories (Kingston, NY). Cohort 1 (Adults) involved 32 female Long-Evans rats that arrived at the vivarium on postnatal day (PND) 62. Cohort 2 (Adolescents) involved 32 female Long-Evans rats that arrived on PND 22. Upon arrival, rats were housed in age-matched pairs in ventilated polycarbonate cages (46 × 24 × 20 cm) under a 12-h light/dark cycle (lights on 08:00, off 20:00) at a controlled ambient temperature of 22-24 °C. Inotiv Teklad® rodent chow and reverse-osmosis tap water were available *ad libitum*. Environmental enrichment (shelter tubes, nesting material, wooden chew blocks) was provided throughout. All procedures conformed to the Canadian Council on Animal Care guidelines and were approved by the Brock University Institutional Animal Care Committee.

### Hormone treatment

**Figure 1:**
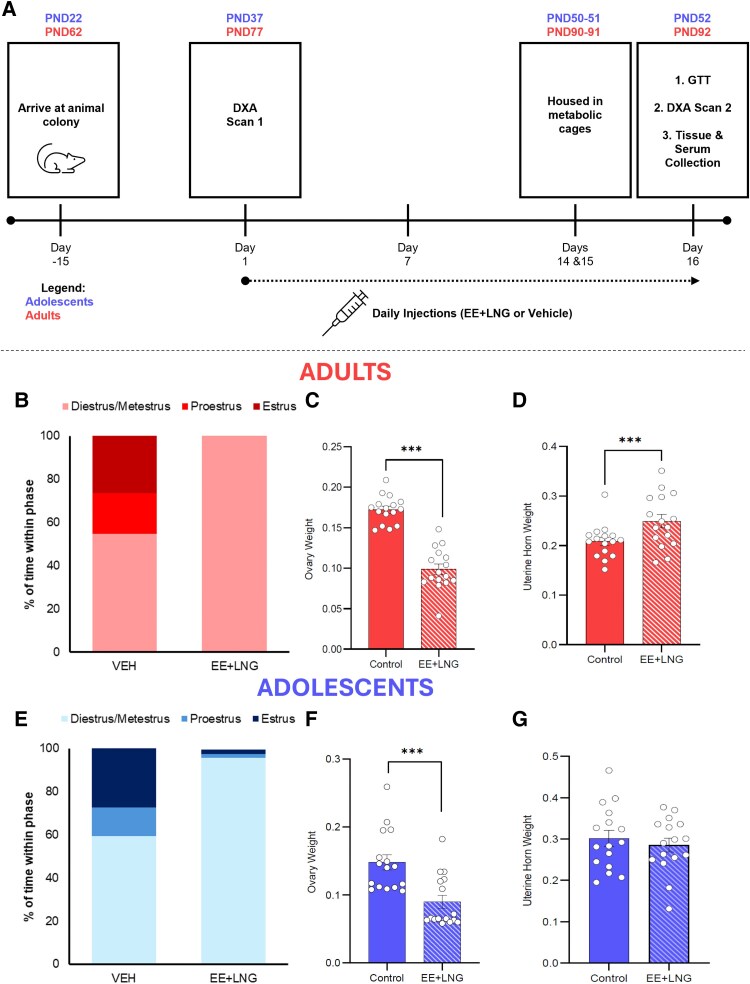
Experimental timeline, estrous cyclicity, and reproductive tissue weights following EE + LNG exposure. (A) Study timeline for adolescent (PND22-52) and adult (PND62-92) cohorts, including dual-energy X-ray absorptiometry (DXA) scans, metabolic cage housing, glucose tolerance testing (GTT), and terminal tissue/serum collection. (B, E) Stacked bar plots depict the proportion of days classified as diestrus/metestrus, proestrus, or estrus during the estrous-tracking window for adults (B) and adolescents (E). (C, F) Ovary weight and (D, G) uterine horn weight are expressed relative to body weight in grams for adults (C, D) and adolescents (F, G). Tissue weights were analyzed within each age by independent-samples *t*-tests comparing EE + LNG vs vehicle. Bars represent mean ± SEM with individual values overlaid; solid bars indicate vehicle and hatched bars indicate EE + LNG. Asterisks denote group differences (*P < .05, **P < .01, ***P < .001). *n* = 16 per treatment per age. EE+LNG, ethnyl estradiol and levonogesterol; GTT, glucose tolerance test; PND, postnatal day.

Ethinyl estradiol (EE; Sigma-Aldrich, PHR1480) and levonorgestrel (LNG; Sigma-Aldrich, PHR1850) were dissolved in 5% ethanol and suspended in sesame oil as the vehicle. We used EE and LNG in combination, which mimics a combination of synthetic hormones found in oral contraceptives most frequently used in North America (5, 6). Most preclinical studies examining the effects of EE and LNG have employed relatively high doses (ie, 10-30 µg EE and 20-125 µg LNG per rat per day). The present study utilized lower, body weight-adjusted doses: 10 µg/kg of EE and 20 µg/kg of LNG administered via subcutaneous injection at a volume of 100 µL/kg for 16 consecutive days (Fig. 1A). This regimen was sufficient to induce marked suppression of endogenous reproductive function, as evidenced by vaginal cytology showing a persistent state of diestrus and a significant reduction in serum luteinizing hormone concentrations (9). These physiological changes closely mimic those observed with HC use in humans. Half of the rats were treated with EE+LNG, and the other half were treated with vehicle.

Injections were administered once daily in the morning between 08:00 and 10:00 for 16 consecutive days, beginning on postnatal day (PND) 37 for adolescent rats and PND 77 for adult rats. In rats, early adolescence begins around postnatal day (PND) 30 and continues until sexual maturity at roughly PND 60 (33), a window characterized by an increase in circulating estrogens and androgens (34-36).

### Estrous cycle tracking

Estrous cyclicity was monitored during the second half of the treatment period using daily vaginal cytology. Vaginal lavages were performed once per day during the final 8 days of the 16-day treatment regimen (treatment days 9-16). Lavages were conducted immediately before daily injections to verify suppression of cyclicity in females treated with EE + LNG and to characterize cycle stage in vehicle-treated females.

Vaginal epithelial cell samples were collected using a standard, noninvasive lavage procedure. Lavage was performed with shallow pipette insertion and gentle aspiration to minimize cervical stimulation and tissue irritation. Briefly, 0.1 mL sterile deionized water was gently pipetted into the vaginal canal, aspirated, and expelled onto a clean glass microscope slide. Slides were air-dried and examined under a light microscope at 20× magnification. Estrous phase was determined based on the relative proportions of cornified epithelial cells, nucleated epithelial cells, and leukocytes, using established cytological criteria (37). The four canonical phases: proestrus, estrus, metestrus, and diestrus were identified. Metestrus and diestrus were combined into a single category for analysis due to their similar cytological profiles. Acyclicity was defined as persistent diestrus-like cytology across consecutive days and the absence of cyclic proestrus/estrus patterns.

### Dual-energy X-ray absorptiometry

Body composition was assessed using a small-animal dual-energy X-ray absorptiometry (DXA) scanner (OsteoSys InSIGHT; Scintica) to noninvasively quantify lean mass and fat mass. Scans were performed on Day 1 and Day 16 of the experimental protocol (see Fig. 1A). Rats were lightly anesthetized with vaporized isoflurane (5% in O_2_) to minimize movement and ensure accurate imaging. Percent lean mass and percent fat mass were calculated relative to total body mass. Absolute lean mass and body mass values obtained via DXA were subsequently used to normalize daily energy expenditure measures (see “Promethion high-definition behavioral phenotyping cages” below).

### Promethion high-definition behavioral phenotyping cages

In the final two days of the study (see Fig. 1A), rats were housed individually for a continuous 48-hour period in Promethion High-Definition Behavioral Phenotyping cages (Sable Systems International, Las Vegas, NV), which allows for real-time, automated monitoring of behavioral and metabolic activity in a home-cage setting. The cages were maintained on a standard 12-hour light/dark cycle. Each cage: (8.6 in (W) × 15.1 in (D) × 9.0 in (H)/21.8 cm × 38.4 cm × 22.9 cm) was equipped with a ceiling-mounted food hopper, a water bottle, and an enclosed climbing-accessible shelter, allowing for the observation of naturalistic behaviors. Photoelectric beam motion detectors were positioned along the X- and Y-axes to track ambulatory movements.

Rats had free access to food, water, and enrichment throughout the testing period. Integrated mass sensors continuously monitored the weight of the food, water, and shelter to quantify consumption and interaction (ie, physical contact or manipulation of the objects). Metabolic and behavioral metrics, including oxygen consumption, respiratory exchange ratio (RER), locomotion, resting behavior, exploratory activity, and object interactions were recorded continuously and averaged in 30-minute intervals using Sable Systems’ proprietary data acquisition software. Sleep time was quantified using duration of immobility of 40 seconds or greater as a surrogate of sleeping as previously described (38). Data were processed and extracted using Macro Interpreter software (v2.48) via the One-Click Macro function (as in (39)).

### Glucose tolerance test

An intraperitoneal glucose tolerance test (GTT) was performed on a subset of rats from each group (*n* = 8) to assess systemic glucose clearance following a glucose load. Rats were fasted for 6 hours before GTT testing, with free access to water. At the end of the fasting period, rats were briefly restrained, and a small incision was made at the distal tip of the tail using a sterile scalpel blade to allow blood sampling. Blood glucose levels were measured using a Freestyle Lite Blood Glucose Monitoring System (Abbott Laboratories), a handheld glucometer designed for small-volume whole blood samples.

Blood samples were collected in duplicate at the following time points: baseline (0 minutes), 15, 30, 45, 60, 90, and 120 minutes following intraperitoneal glucose administration. Glucose was administered at a dose of 2 g/kg body mass as a sterile 20% D-glucose solution (w/v) in saline, injected intraperitoneally. The area under the curve for glucose values was calculated to quantify total glycemic response over the 120-minute testing period (as in (40)).

### Tissue collection

Rats were euthanized by nonsurvival surgery under isoflurane anesthesia, in accordance with institutional animal care and use guidelines.

#### Adipose tissue collection

Inguinal white adipose tissue (iWAT) was collected from the bilateral subcutaneous inguinal depots, and brown adipose tissue (BAT) was dissected from the interscapular region. Gonadal white adipose tissue (gWAT) was harvested from the periovarian region, and the ovaries and uterine horns were collected. Tissues were carefully excised, trimmed of excess connective tissue, and weighed using an analytical balance. Tissues were stored at −80 °C until processing for analysis.

#### Brain tissue collection

Animals were rapidly decapitated and brains were quickly removed. Brains were flash frozen in precooled isopentane chilled on dry ice until fully frozen, within 1 minute of decapitation, individually wrapped in foil, and stored at −80 °C until sectioning and tissue processing.

### Tissue analyses

#### Western blotting

Adipose tissue samples were homogenized using a FastPrep FP120 Tissue Homogenizer (Savant) in NP-40 lysis buffer at a 1:3 ratio of tissue mass (mg) to lysis buffer volume (µL) for white adipose depots and a 1:5 ratio for brown adipose tissue (BAT). The lysis buffer consisted of 10 mL NP-40 lysis buffer supplemented with 34 µL phenylmethylsulfonyl fluoride and 50 µL protease inhibitor. Homogenates were centrifuged at 4 °C for 5 minutes at 1500 × g, and the middle aqueous layer was collected; the pellet and upper lipid layer were discarded. Protein concentrations were determined using a bicinchoninic acid (BCA) assay. Samples were then prepared to contain equal protein concentrations in 2× Laemmli buffer and were denatured at 100 °C for 5 minutes. Brown adipose tissue samples were loaded at 5 µg protein per lane and gonadal white adipose tissue (gWAT) samples at 20 µg protein per lane onto 10% SDS–PAGE gels, electrophoresed for 90 minutes at 120 V, and wet-transferred onto 0.45-µm nitrocellulose membranes. Membranes were stained with Ponceau S solution (BioShop) to verify even protein loading and subsequently destained with TBST (tris-buffered saline, 0.1% Tween 20) washes (3 × 5 minutes). Membranes were blocked in 5% nonfat dry milk in TBST for 1 hour and incubated overnight at 4 °C with primary antibody against uncoupling protein 1 (UCP1) (Abcam; cat. no. ab10983) at 1:1000. Membranes were rinsed with TBST and incubated for 1 hour at room temperature with donkey antirabbit secondary antibody at 1:5000, then washed again and imaged. Chemiluminescent signal was detected on a Bio-Rad ChemiDoc Touch Imaging System (cat. no. 1708370) using Western Lightning ECL (cat. no. NEL105001EA). Densitometric analyses were performed in Bio-Rad Image Lab Software, and target protein levels were normalized to the corresponding Ponceau S loading control. Uncoupling protein 1 was not quantified in inguinal white adipose tissue (iWAT) because prior studies in female rodents indicate that iWAT UCP1 abundance often falls below reliable detection limits (41, 42), making measurement in this depot frequently uninformative.

#### Quantitative polymerase chain reaction

##### Hypothalamic tissue punches

Brains were cryosectioned at −20 °C. A prechilled 1.0-mm hollow biopsy punch (Integra™ Miltex™ Biopsy Punches; cat. no. 1260402) was used to collect micropunches targeting the arcuate nucleus of the hypothalamus (0.24 mm to −3.48 mm relative to Bregma), guided by Paxinos and Watson stereotaxic coordinates. Punches were collected into prechilled RNase-free tubes and stored at −80 °C until processing.

##### RNA isolation

Hypothalamic punches were homogenized in 500 µL TRIzol Reagent (Thermo Fisher Scientific; cat. no. 15596026). Following phase separation with chloroform (Fisher; cat. no. C2981), RNA was isolated from the aqueous phase using the RNeasy Mini Kit (Qiagen; cat. no. 74104) according to the manufacturer's instructions. RNA concentration was quantified using an Implen NanoPhotometer P300.

##### Quantitative PCR

First-strand cDNA was synthesized from 2.5 µg total RNA using SuperScript IV VILO (Thermo Fisher Scientific; cat. no. 11756050). Gene expression was quantified using TaqMan Gene Expression Assays (Thermo Fisher Scientific) with TaqMan Fast Advanced Master Mix (cat. no. 4444556) and probes for Agrp (*Rn01431703_g1*), Npy (Rn01410145_m1), Pomc (*Rn00595020_m1*), Cartpt (*Rn00567382_m1*), and the reference genes Rpl13a (Rn00821946_g1) and Tbp (Rn01455646_m1). Quantitative polymerase chain reaction (PCR) was performed on a Bio-Rad CFX96 system. All reactions were run in triplicate, and mean Cq values were used for analysis. Target gene expression was normalized to the geometric mean of the two reference genes (Rpl13a and Tbp) by calculating the average reference Cq across the two genes for each sample (equivalent to the geometric mean in expression space). Relative expression was determined using the ΔΔCq method.

### Serum analyses

#### Enzyme-linked immunosorbent assays

Serum samples were obtained from trunk blood collected immediately following rapid decapitation during terminal tissue collection performed under isoflurane anesthesia. At terminal sampling, EE + LNG-treated rats exhibited persistent diestrus-like cytology, whereas vehicle-treated rats represented a mixture of naturally occurring estrous stages (see Table 1). Serum was used to quantify circulating luteinizing hormone (LH) and 17β-estradiol (E2) using commercially available rat enzyme-linked immunosorbent assays (ELISA) kits (Thermo Fisher Scientific; LH: cat. no. EEL122/RRID: AB_3741196; E2: cat. no. EELR015/RRID: AB_3741193). All assays were performed according to the manufacturer's protocols and read on a microplate reader at 450 nm. Samples were run in duplicate and mean values were used for analysis. Intraassay coefficient of variation (CV%) was 3.75% for LH and 5.11% for E2. The lower limits of detection (LLOD; manufacturer-reported analytical sensitivity) were 0.94 mIU/mL for LH and 1.17 pg/mL for E2. Samples falling below the lower limit of quantification (LLOQ) or above the upper limit of quantification (ULOQ) were excluded from statistical analyses. <5% of samples were below the LLOQ and excluded from the statistical analysis. Exclusions included: one rat from the LH analysis (adolescent vehicle group), and three rats from the E2 analysis (adolescent vehicle; *n* = 1, adolescent EE + LNG; *n* = 1, adult vehicle; *n* = 1). No significant cross-reactivity was reported by the manufacturer.

**Table 1:** Distribution of estrous cycle phases on the final day of the experiment shown separately by treatment group and age cohort

	Adults		Adolescents	
	Cycle phase	*n*	Cycle phase	*n*
**Control**	Diestrus/metestrus	10	Diestrus/metestrus	11
	Proestrus	1	Proestrus	1
	Estrus	5	Estrus	4
**EE** **+** **LNG**	Diestrus/metestrus	16	Diestrus/metestrus	16
	Proestrus	0	Proestrus	0
	Estrus	0	Estrus	0

#### Multiplex immunoassay for metabolic markers

Serum samples were obtained via trunk blood collection from a subset of rats (*n* = 8 per group) after a 6-hour fasting period immediately before euthanasia. Serum was used to quantify metabolic biomarkers using the U-PLEX® Metabolic Group 1 (rat) multiplex assay (Meso Scale Discovery, K15316K-1), which enables simultaneous detection of seven analytes relevant to metabolic function. These include C-peptide, ghrelin (active), glucagon, GLP-1 (active), insulin, leptin, and PYY (total). Samples were run in duplicate, and mean values were used for analysis, according to the manufacturer's protocol. Intraassay coefficient of variation (CV%) remained below 10%. The lower limits of detection (LLOD) for each analyte were as follows: C-peptide, 220 pg/mL; ghrelin (active), 13 pg/mL; GLP-1 (active), 0.14 pM; glucagon, 0.13 pM; insulin, 3.0 µIU/mL; leptin, 11 pg/mL; and PYY (total), 1.1 pg/mL. <5% of samples were below the LLOQ and excluded from the statistical analysis. One rat from the adolescent control group was excluded from analysis because of poor analyte detectability. No significant cross-reactivity between analytes was reported by the manufacturer.

### Statistical analyses

Statistical analyses were performed using IBM SPSS Statistics (Version 29), and figures were generated in GraphPad Prism (Version 8.4.3). Because most primary outcomes (ie, body weight, fat mass, lean mass, food intake) scale with body size, modeling Age as a factor would have absorbed variance and generated Age × Treatment interactions driven by baseline size differences rather than pharmacologic effects. We therefore analyzed adolescents and adults separately, a physiologically grounded approach that yields interpretable within-age HC effects.

Daily changes in body weight were analyzed using two-factor mixed-design repeated-measures ANOVAs with Day 1-16 as the within-subjects factor and Treatment (EE + LNG vs Vehicle) as the between-subjects factor, conducted separately for adolescents and adults. Dual-energy X-ray absorptiometry outcomes were analyzed using two-way repeated-measures ANOVA with Time (Scan 1 vs Scan 2) as the within-subjects factor and Treatment (EE + LNG vs vehicle) as the between-subjects factor. Luteinizing hormone and E2 concentrations were also analyzed using a two-way repeated-measures ANOVA with Hormone (LH, E2) as the within-subject factor and Treatment (EE + LNG vs vehicle) as the between-subject factor.

For hypothalamic qPCR data, mean Cq values (averaged across technical triplicates) were used to calculate ΔCq for each target gene (AgRP, NPY, POMC, and CART) by subtracting a reference value derived from the geometric mean of Rpl13a and Tbp. Gene expression was analyzed separately by gene, using two-tailed independent-samples Student's *t*-tests to compare EE + LNG-treated rats with vehicle-treated rats on ΔCq values. Relative expression was additionally expressed as fold-change using the ΔΔCq method, with vehicle serving as the calibrator within each age group (fold-change = 2^−ΔΔCq). All other comparisons between treatment groups (eg, food and water intake, metabolic cage activity, serum hormone levels, glucose tolerance test area under the curve, and postmortem tissue weights, adipose tissue UCP1 content) were assessed using two-tailed independent-samples *t*-tests.

Estrous cycling was evaluated separately within adolescents and adults using a predefined *cycling* criterion based on vaginal cytology across the tracking window. Rats were classified as cyclic if they exhibited ≥2 estrus days or ≥1 proestrus day and ≥1 estrus day. Group differences in the proportion of cyclic animals (vehicle vs EE + LNG) were assessed using Fisher's exact tests (*α* = .05).

Effect sizes are reported as Cohen's *d* for *t*-tests and partial eta squared (*η*_p_^2^) for ANOVAs, where applicable. Post hoc tests used Newman-Keuls comparisons. A priori comparisons used Bonferroni corrections and were two-tailed unless specified. Significance threshold was set at *P* < .05.

## Results

### EE + LNG treatment produces acyclicity, reduced ovarian weight and Serum LH and estradiol, regardless of age

#### EE + LNG-treatment led to a state of acyclicity in both age groups

*Adults*: Vehicle-treated adults were more likely to meet criteria for *cycling* than EE + LNG-treated adults (16/16 vs 0/16, respectively; Fisher's exact, *P* < .001) (data summarized in Fig. 1B).

*Adolescents*: Similar to adults, vehicle-treated adolescents met criteria for *cycling* compared with EE + LNG-treated adolescents (16/16 vs 0/16, respectively; Fisher's exact, *P* < .001) (data summarized in Fig. 1E).

#### EE + LNG-treatment in adulthood and adolescence reduced ovary weight but only increased uterine horn weight in adults

Reproductive organ weights were normalized to body mass on the day of collection.

*Adults*: Relative to vehicle, EE + LNG rats had lighter ovaries (*t*_30_ = −9.55, *P* < .001, *d* = −3.4; Fig. 1C) and heavier uterine horns (*t*_30_ = 2.56, *P* = .016, *d* = 0.90; Fig. 1D; Table 2).

**Table 2:** Summary of anthropometric results

	Adults				Adolescents			
Variable	Control	EE + LNG	*P*	*d*	Control	EE + LNG	*P*	*d*
Body weight, day 1 (g)	248.81 ± 5.97	247.68 ± 6.16	.897	0.05	117.00 ± 3.15	118.31 ± 2.69	.753	−0.11
Body weight, day 16 (g)	274.69 ± 5.96	241.00 ± 5.56	<.001***	1.46	192.50 ± 3.40	159.38 ± 2.65	<.001***	2.72
% body weight change	10.52 ± 0.74	−2.54 ± 1.11	<.001***	3.46	65.51 ± 3.18	35.11 ± 1.67	<.001***	2.99
Inguinal adipose tissue	1.61 ± 0.14	0.85 ± 0.08	<.001***	1.67	1.08 ± 0.05	0.84 ± 0.05	.001**	1.20
Gonadal adipose tissue	2.25 ± 0.14	0.75 ± 0.09	<.001***	3.19	0.94 ± 0.09	0.38 ± 0.04	<.001***	2.01
Brown adipose tissue	0.21 ± 0.01	0.17 ± 0.01	.038*	1.00	0.25 ± 0.02	0.24 ± 0.02	.669	0.13
Ovarian tissue	0.17 ± 0.00	0.10 ± 0.01	<.001***	2.47	0.15 ± 0.01	0.09 ± 0.01	<.001***	1.50
Uterine horn tissue	0.21 ± 0.01	0.25 ± 0.01	.008**	−1.00	0.30 ± 0.01	0.29 ± 0.02	.536	0.16

Anthropometric data for adult and adolescent rats treated with EE + LNG or vehicle. Values are presented as means ± SEM. Tissue weights are expressed as a percentage of total body weight (BW). Group differences were evaluated using post hoc comparisons. *n* = 16 per group. Significant differences are indicated as follows and in bold: *P < .05, **P < .01, ***P < .001. Effect sizes are reported as Cohen's *d*.

*Adolescents*: Relative to vehicle, EE + LNG rats had lighter ovaries (*t*_30_ = −3.95, *P* < .001, *d* = −1.4; Fig. 1F), whereas uterine horn weight did not differ between EE + LNG-treated and vehicle-treated rats (*P* = .536; Fig. 1G; Table 2).

#### Serum LH and E2 concentrations were lower in EE + LNG-treated rats than in vehicle-treated rats in both age groups

*Adults*: LH and E2 levels were significantly lower in EE + LNG-treated adults than vehicle-treated adults (one tailed, LH: *P* < .001; E2: *P* = .041, *d* = 0.67; Table 3; main effect of Treatment, *F*_1, 27_ = 5.76, *P* = .024, *η*_p_^2^ = 0.176). There were no other significant main or interaction effects (*P* > .264).

**Table 3:** Summary of serum luteinizing hormone and 17β-estradiol concentrations

	Adults				Adolescents			
Hormone (units)	Control	EE + LNG	*P*	*d*	Control	EE + LNG	*P*	*d*
Luteinizing hormone (mIU/mL)	30.33 ± 1.40 (18.71-46.84)	23.30 ± 1.36 (8.79-30.67)	< .001***	1.27	31.48 ± 1.97 (18.36-46.83)	25.71 ± 1.89 (8.56-38.70)	.021*	0.76
17β-estradiol (pg/mL)	35.27 ± 10.65 (12.03-139.32)	15.16 ± 1.76 (7.52-33.29)	.041*	0.67	22.92 ± 2.97 (9.84-54.38)	17.36 ± 1.62 (2.90-26.48)	.056	0.62

Serum luteinizing hormone and 17β-estradiol concentration by age and treatment. Group differences were evaluated using post hoc comparisons. Group sizes were *n* = 16 per group, except for LH, where the adolescent control group was *n* = 15, and for E2, where group sizes were reduced following exclusions (adolescent control *n* = 15, adolescent EE + LNG *n* = 15, and adult control *n* = 15). Values are presented as mean ± SEM with ranges in parentheses. Significant differences are indicated as follows and in bold: *P < .05, ***P < .001. Effect sizes are reported as Cohen's *d*.

*Adolescents*: LH was significantly lower in EE + LNG-treated adolescents (one tailed, *P* = .0218, *d* = 0.76) and there was a trend for E2 to be lower in the EE + LNG adolescents compared with the vehicle-treated adolescents (one-tailed *P* = .056, *d* = 0.62; main effect of Treatment: *F*_1, 25_ = 15.64, *P* < .001, *η*_p_^2^ = 0.376). There were no other main or interaction effects (*P* > .624)

### EE + LNG treatment reduces body mass accrual and food intake in both age groups

**Figure 2:**
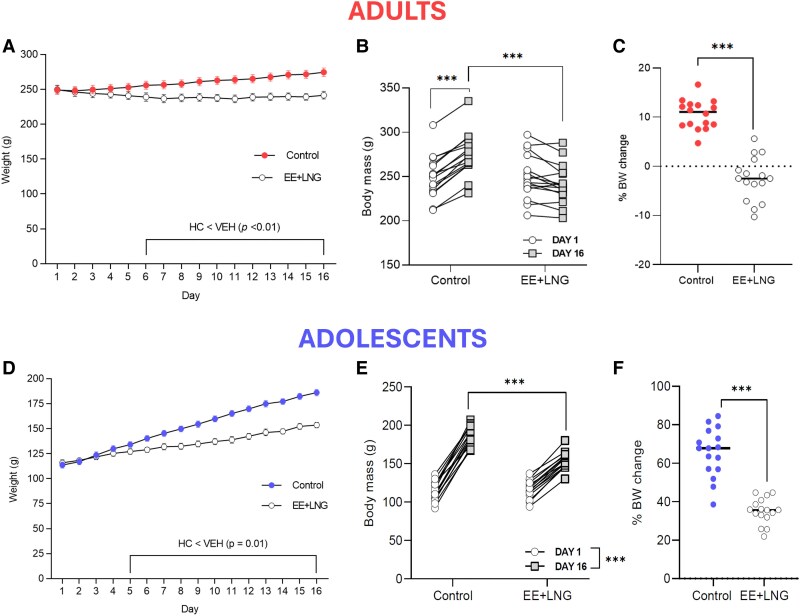
EE + LNG attenuates body-weight gain across 16 days in adult and adolescent female rats. (A-C) Adults and (D-F) adolescents. (A, D) Daily body weight (g) across Days 1-16 (mean ± SEM; Vehicle (VEH), filled symbols; EE + LNG, open symbols). Within each age group, body weight was analyzed using a two-factor mixed-design repeated-measures ANOVA with Day (1-16) as the within-subject factor and Treatment (EE + LNG vs Vehicle) as the between-subject factor. The bracketed interval in (A, D) denotes days on which EE + LNG differed from Vehicle based on Bonferroni-adjusted simple-effects comparisons of Treatment within each day following the Day × Treatment interaction. (B, E) Within-animal body mass (g) at Day 1 and Day 16 (lines connect individual rats). (C, F) Percent change in body mass from Day 1 to Day 16. Asterisks denote statistically significant differences for the comparisons indicated by brackets (***P < .001). *n* = 16 per treatment per age.

*Adults*: EE+LNG-treated rats accrued less weight across the 16 days than vehicle controls (Day × Treatment interaction: *F*_15,450_ = 114.16, *P* < .001, *η*_p_^2^ = 0.792; Fig. 2A). EE + LNG-treated rats weighed significantly less from Day 6 through Day 16 (all *P*'s < .035). Vehicle-treated rats gained body mass from Day 1 to Day 16 (*P* < .001), rats treated with EE + LNG did not (*P* > .5; Fig. 2C; Table 2).

**Figure 3:**
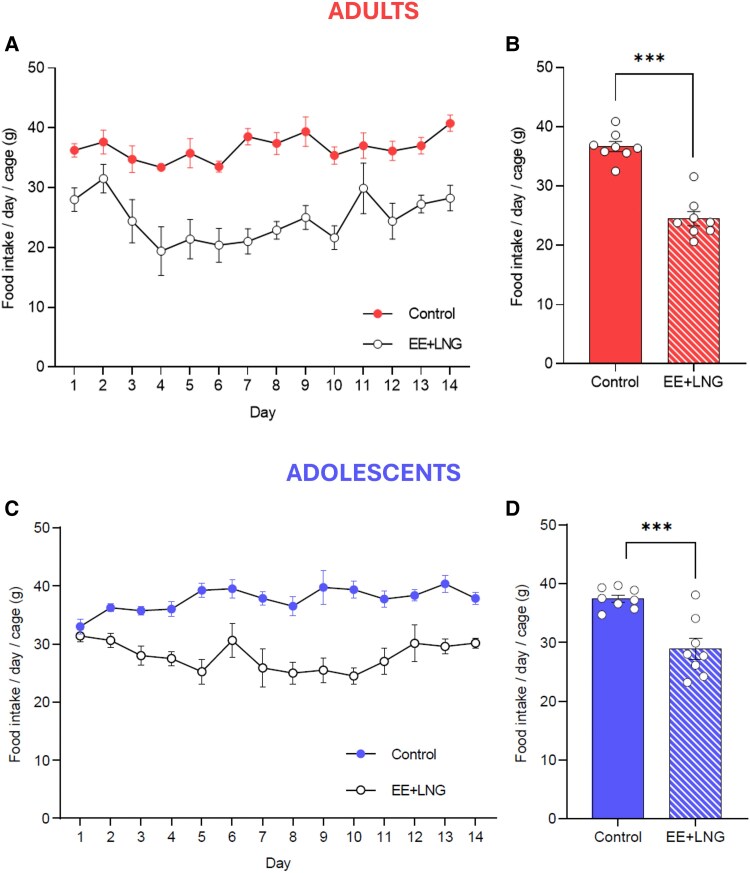
EE + LNG reduces daily and average food intake in adult and adolescent rats. (A-B) Adults and (C-D) Adolescents. (A, C) Cage-level food intake (g/day/cage) measured across 14 consecutive days of treatment (mean ± SEM), shown separately for vehicle (filled symbols/lines) and EE + LNG (open symbols/lines). (B, D) Mean daily food intake averaged across the 14-day period (points represent individual cages; bars represent mean ± SEM). Mean daily intake was analyzed within each age group using two-tailed independent-samples *t*-tests comparing EE + LNG vs vehicle at the cage level. Asterisks denote a significant treatment difference for the bracketed comparison (***P < .001). *n* = 8 cages per treatment per age (2 rats/cage).

EE+LNG-treated rats consumed less food when averaged across the 16-day treatment period compared with vehicle-treated rats, (*t*_30_ = −12.18, *P* < .001, *d* = −4.30; Fig. 3A and 3B).

*Adolescents*: EE + LNG-treated adolescent rats accrued less weight across the 16 days than vehicle controls (Day × Treatment interaction: *F*_15,450_ = 100.77, *P* < .001, *η*_p_^2^ = 0.771; Fig. 2D). Although there were no significant differences in body weight at baseline (*P* = .753), HC adolescent rats weighed less than controls by Day 5 through Day 16 (all *P*'s < .01). Both vehicle-treated rats (*P* < .001) and EE + LNG-treated rats (*P* < .001) gained body mass across days (Fig. 2F; Table 2).

EE + LNG-treated rats consumed less food when averaged across the 16-day treatment period compared with vehicle-treated rats (*t*_28_ = −6.07, *P* < .001, *d* = −2.22; Fig. 3C and 3D).

### EE + LNG reduces fat-mass accrual and adipose depot size while preserving lean-mass percentage in adults and adolescents

**Figure 4:**
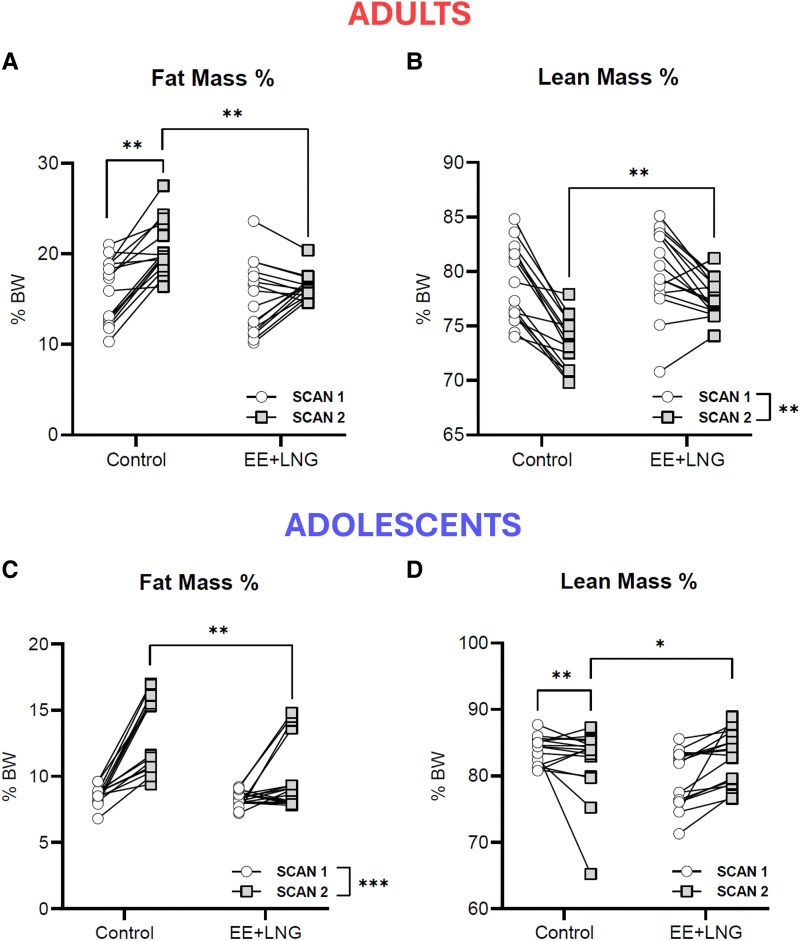
EE + LNG alters DXA-derived body composition in adults and adolescents. (A-B) Adults and (C-D) Adolescents. DXA was performed at Scan 1 (baseline) and Scan 2 (post-treatment), and fat mass (A, C) and lean mass (B, D) are expressed as % body weight (%BW). Circles denote Scan 1 and squares denote Scan 2; lines connect repeated measures from the same rat. Within each age group, outcomes were analyzed using two-factor mixed-design repeated-measures ANOVAs with Time (Scan 1 vs Scan 2) as the within-subject factor and Treatment (EE + LNG vs Vehicle) as the between-subject factor. Where brackets connect Scan 1 to Scan 2 within a treatment group, asterisks denote post hoc tests of Time within Treatment. Where brackets connect Vehicle vs EE + LNG at Scan 2, asterisks denote post hoc tests of Treatment within Time (endpoint group differences). Where asterisks appear beside the Scan 1 and Scan 2 legend key, they denote a significant main effect of Time from the omnibus ANOVA. Asterisks indicate the level of significance for the comparison shown (*P < .05, **P < .01). *n* = 16 per treatment per age.

*Adults*: There were no significant treatment group differences in adults for fat and lean mass percentage on Scan 1 (fat mass: *P* = .563; lean mass: *P* = .332; Fig. 4A and 4B). On Scan 2, EE + LNG-treated adult rats exhibited lower fat mass percentage compared with vehicle-treated rats (*P* < .001; Scan × Treatment interaction, *F*_1,30_ = 10.08, *P* = .003, *η*_p_^2^ = 0.251; Fig. 4A). Fat mass percentage increased from Scan 1 to Scan 2 in the vehicle-treated group (*P* < .001; Fig. 4A), and not in the EE + LNG-treated group (*P* = .093). On Scan 2, EE + LNG-treated adult rats had a higher lean mass percentage than did vehicle-treated rats (*P* < .001; Scan × Treatment interaction, *F*_1,30_ = 8.15, *P* = .008, *η*_p_^2^ = 0.214; Fig. 4B). Lean mass percentage decreased from Scan 1 to Scan 2 in both groups (all *Ps* = .001; Fig. 4B).

**Figure 5:**
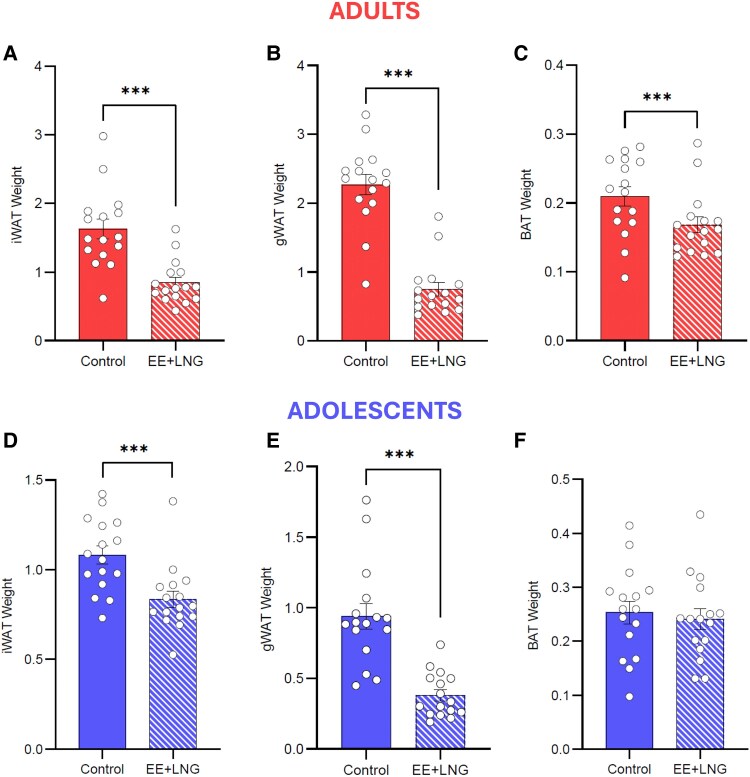
EE + LNG reduces white adipose depots in adults and adolescents, with age-specific effects on BAT mass. (A-C) Adults and (D-F) Adolescents. Postmortem adipose depot weights are shown normalized to body weight in grams for (A, D) inguinal white adipose tissue (iWAT), (B, E) gonadal white adipose tissue (gWAT), and (C, F) brown adipose tissue (BAT). Within each age group, depot weights were analyzed using two-tailed independent-samples *t*-tests comparing EE + LNG vs vehicle. Bars represent mean ± SEM with individual values overlaid; solid bars indicate vehicle and hatched bars indicate EE + LNG. Asterisks denote statistically significant treatment differences for the bracketed comparisons (***P < .001). *n* = 16 per treatment group.

**Figure 6:**
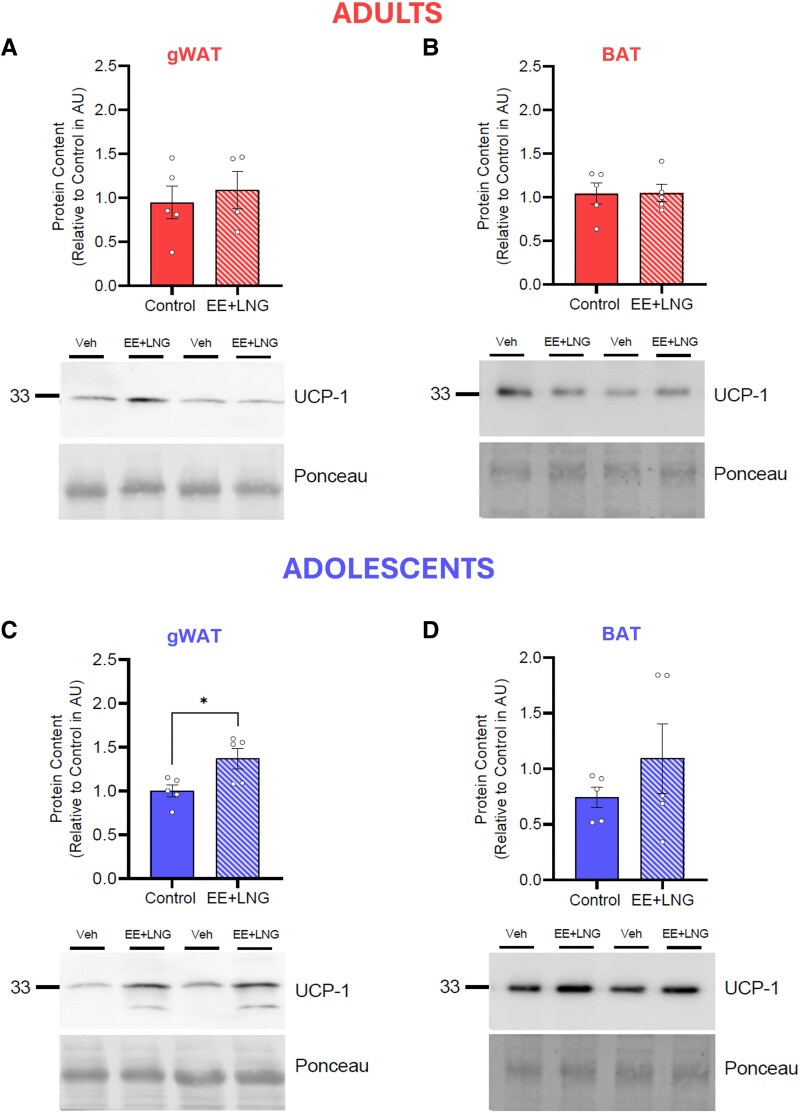
UCP1 protein content in gWAT and BAT following EE + LNG exposure in adults and adolescents. (A-B) Adults and (C-D) adolescents. UCP1 protein content was quantified by western blotting in (A, C) gonadal white adipose tissue (gWAT) and (B, D) brown adipose tissue (BAT) after 16 days of EE + LNG or vehicle treatment. UCP1 band intensity was quantified by densitometry and normalized to the corresponding Ponceau S loading control. Bars represent mean ± SEM with individual values overlaid; solid bars indicate vehicle and hatched bars indicate EE + LNG. Within each age group, treatment effects were tested using two-tailed Welch's independent-samples *t*-tests separately for gWAT and BAT (four tests total). Asterisks denote statistically significant treatment differences for the bracketed comparisons (*P < .05). *n* = 5 per group.

Compared with vehicle-treated rats, EE + LNG treatment markedly reduced the relative size of inguinal white adipose tissue depots (iWAT; *t*_30_ = −4.76, *P* < .001, *d* = −1.7; Fig. 5A), gonadal white adipose tissue depots (gWAT; *t*_30_ = −8.49, *P* < .001, *d* = −3.0; Fig. 5B) and brown adipose tissue depots (BAT; *t*_30_ = −2.17, *P* = .038, *d* = −0.77; Fig. 5C). Neither gWAT UCP1 nor BAT UCP1 differed by treatment (gWAT: *t*_6.43_ = −0.51, *P* = .629, *d* = −0.34; Fig. 6A; BAT: *t*_7.65_ = −0.04, *P* = .970, *d* = −0.02; Fig. 6B).

*Adolescents*: There were no treatment group differences in adolescent rats in fat mass percentage or lean mass percentage on Scan 1 (fat mass: *P* = .395; lean mass: *P* = .265; Fig. 4C-4D). Ethinyl estradiol + LNG-treated rats exhibited lower fat mass percentage compared with vehicle-treated rats on Scan 2 (*P* = .002; Scan × Treatment interaction, *F*_1,30_ = 10.86, *P* = .003, *η*_p_^2^ = 0.27; Fig. 4C). Fat mass percentage increased from Scan 1 to Scan 2 in both groups (*P*s <.05; Fig. 4C). Ethinyl estradiol + LNG-treated adolescent rats had a higher lean mass percentage than vehicle-treated rats by Scan 2 (*P* = .029; Scan × Treatment interaction, *F*_1,30_ = 4.92, *P* = .03, *η*_p_^2^ = 0.141; Fig. 4D). Lean mass percentage decreased from Scan 1 to Scan 2 in the vehicle-treated group (*P* = .008; *d* = 1.34), whereas no significant change was observed in the EE + LNG group (*P* = .753; Fig. 4D).

Compared with vehicle-treated adolescent rats, EE + LNG treatment in adolescence markedly reduced the relative size of inguinal white adipose tissue depots (iWAT; *t*_30_ = −3.59, *P* = .001, *d* = −1.3; Fig. 5D) and gonadal white adipose tissue depots (gWAT; *t*_30_ = −5.68, *P* < .001, *d* = −2.0; Fig. 5E). However, BAT did not significantly differ between treatment groups (*P* = .669; Fig. 5F). In adolescents, gWAT UCP1 was significantly higher in EE + LNG-treated rats than vehicle-treated rats (*t*_6.52_ = −2.72, *P* = .032, *d* = −1.72; Fig. 6C). In contrast, BAT UCP1 did not significantly differ by Treatment (*t*_4.68_ = −1.08, *P* = .33, *d* = −0.68; Fig. 6D). (Adipose tissue weight data are summarized in Table 2).

### EE + LNG in adulthood and adolescence increases energy expenditure without affecting cage ambulation

**Figure 7:**
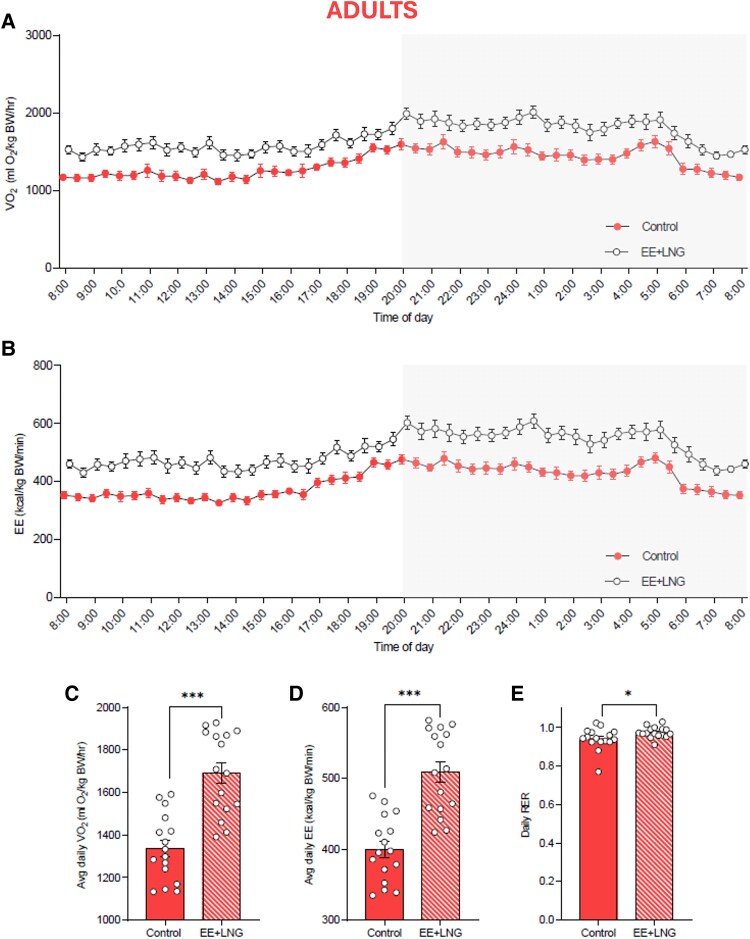
EE + LNG increases oxygen consumption and energy expenditure in adult rats. (A-B) Hourly profiles of whole-body oxygen consumption (VO_2_; mL O_2_·kg BW^−1^·min^−1^) and energy expenditure (EE; kcal·kg BW^−1^·min^−1^) across the light–dark cycle (white background = lights on; gray background = lights off). Vehicle controls are shown in filled symbols/lines and EE + LNG-treated rats in open symbols/lines; values represent mean ± SEM at each time point. (C-D) Mean daily VO_2_ and mean daily EE averaged across the recording interval (points = individual rats; bars = mean ± SEM). (E) Mean daily respiratory exchange ratio (RER; unitless) averaged across the recording interval. Daily mean outcomes (C-E) were analyzed using two-tailed independent-samples *t*-tests comparing EE + LNG vs vehicle in adults. Asterisks denote statistically significant treatment differences for the bracketed comparisons (*P < .05, **P < .01, ***P < .001). *n* = 16 per group.

*Adults*: EE + LNG-treated adult rats had a higher total oxygen consumption (*VO_2_*; *t*_30_ = 5.695, *P* < .001, *d* = 2.01; Fig. 7A and 7C), total daily energy expenditure (*t*_30_ = 5.179, *P* < .001, *d* = 1.831; Fig. 7B and 7D), and respiratory exchange ratio (*t*_30_ = 2.181, *P* = .037, *d* = .77; Fig. 7E) compared with vehicle-treated controls.

EE + LNG-treated adult rats also drank more water relative to vehicle-treated controls (*t*_30_ = 2.16, *P* = .039, *d* = .76). The two groups did not differ in cage ambulation (*P* = .457), food intake (*P* = .162), or percentage of time spent asleep (*P* = .837; Table 4).

**Table 4:** Summary of results from behavioral phenotyping metabolic cages

	Adults				Adolescents			
Variable	Control	EE + LNG	*P*	*d*	Control	EE + LNG	*P*	*d*
**Behavioral Measures**								
Food intake (g)	14.44 ± 2.63	9.95 ± 1.70	.162	0.51	13.77 ± 1.99	9.71 ± 1.51	.116	0.57
Water intake (mL)	22.75 ± 4.98	39.97 ± 6.23	.039*	−0.76	24.76 ± 2.77	34.25 ± 3.77	.051	−0.72
Time spent in house (%)	21.46 ± 6.95	9.49 ± 4.06	.147	0.53	45.87 ± 6.20	27.40 ± 6.02	.041*	0.76
Time spent sleeping (%)	47.41 ± 2.40	46.69 ± 2.51	.837	0.07	52.75 ± 3.11	53.02 ± 3.12	.952	−0.02
Cage ambulation (meters)	371.74 ± 24.51	348.37 ± 19.05	.457	0.27	243.32 ± 12.91	231.94 ± 8.76	.471	0.26
**Metabolic measures**								
VO_2_ (total) (mL O_2_/kg BW/hour)	1334.15 ± 39.01	1687.29 ± 48.20	<.001***	−2.01	1679.49 ± 36.35	2398.45 ± 53.78	<.001***	−3.92
VO_2_ (light)	1232.84 ± 34.52	1568.61 ± 46.79	<.001***	−2.04	1552.45 ± 35.95	2283.10 ± 49.57	<.001***	−4.22
VO_2_ (dark)	1431.41 ± 44.81	1801.23 ± 52.71	<.001***	−1.89	1801.45 ± 42.26	2509.18 ± 60.90	<.001***	−3.38
Energy expenditure (total) (kcal/kg BW/hour)	0.1106 ± 0.004	0.1419 ± 0.005	<.001***	−1.73	0.1444 ± 0.003	0.2069 ± 0.005	<.001***	−3.79
Energy expenditure (light)	0.1013 ± 0.003	0.1306 ± 0.004	<.001***	−2.07	0.1288 ± 0.003	0.1894 ± 0.004	<.001***	−4.29
Energy expenditure (dark)	0.1188 ± 0.004	0.1506 ± 0.006	<.001***	−1.56	0.1581 ± 0.003	0.2206 ± 0.006	<.001***	−3.29
Respiratory exchange ratio	0.9400 ± 0.014	0.9750 ± 0.007	.037*	−0.79	0.9669 ± 0.008	0.9925 ± 0.007	.024*	−0.85

Behavioral and metabolic outcomes in adult and adolescent rats treated with EE + LNG or vehicle, assessed using metabolic phenotyping cages. Values are presented as means ± SEM. Group differences were evaluated using post hoc comparisons. Both are presented as total values, and separately for the light and dark cycles. *n* = 16 per group. Significant differences are indicated as follows and in bold: *P < .05, ***P < .001. Effect sizes are reported as Cohen's *d*.

**Figure 8:**
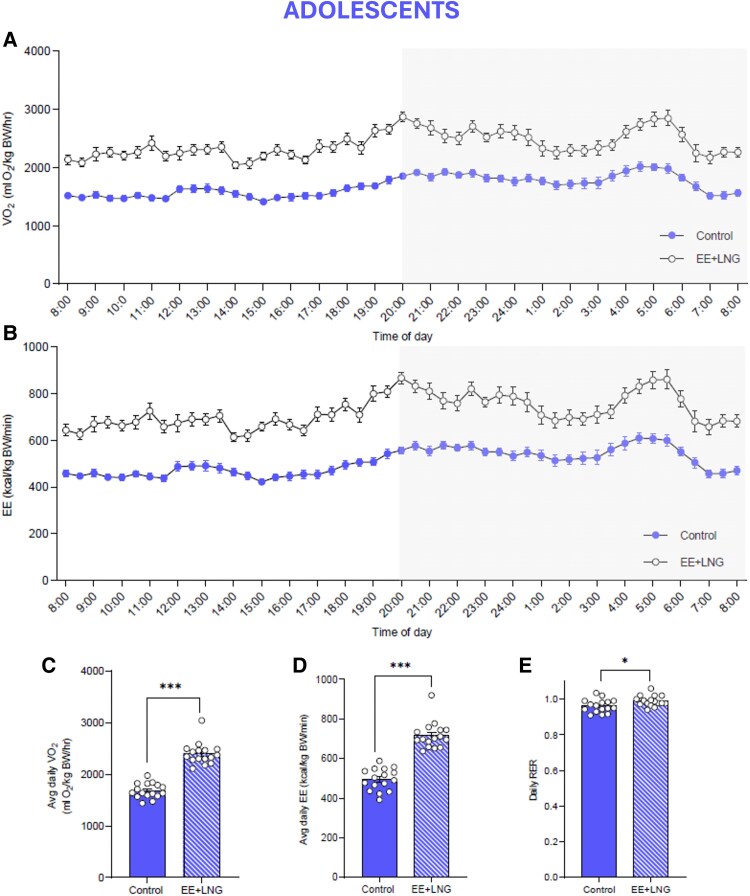
EE + LNG increases oxygen consumption and energy expenditure in adolescent rats. (A-B) Hourly profiles of oxygen consumption (VO_2_; mL O_2_·kg BW^−1^·min^−1^) and energy expenditure (EE; kcal·kg BW^−1^·min^−1^) across the light–dark cycle (white background = lights on; gray background = lights off). Vehicle controls are shown in filled symbols/lines and EE + LNG-treated rats in open symbols/lines; values represent mean ± SEM at each time point. (C-E) Mean daily VO_2_, mean daily EE, and mean daily respiratory exchange ratio (RER; unitless) averaged across the recording interval (points = individual rats; bars = mean ± SEM). Group differences in daily mean outcomes (C-E) were tested in adolescents using two-tailed independent-samples *t*-tests comparing EE + LNG vs vehicle. Asterisks denote statistically significant treatment differences for the bracketed comparisons (*P < .05, **P < .01, ***P < .001). *n* = 16 per group.

*Adolescents*: EE + LNG-treated adolescent rats had a higher total oxygen consumption (VO_2_; *t*_30_ = 9.55, *P* < .001, *d* = 3.37; Fig. 8A and 8C), total daily energy expenditure (*t*_30_ = 11.15, *P* < .001, *d* = 3.94; Fig. 8B and 8D), and respiratory exchange ratio (*t*_30_ = 2.38, *P* = .024, *d* = .84; Fig. 8E) compared with vehicle-treated controls. Ethinyl estradiol + LNG-treated adolescent rats also spent a lower percentage of time within the house zone of the metabolic cage (*t*_30_ = −2.14, *P* = .041, *d* = −0.76). There were no significant differences between groups in cage ambulation (*P* = .471), food (*P* = .116) or water intake (*P* = .051), or percentage of time spent asleep (*P* = .952; Table 4).

### EE + LNG alters different metabolic markers in adults and adolescent, and improves glucose tolerance in adults only

**Figure 9:**
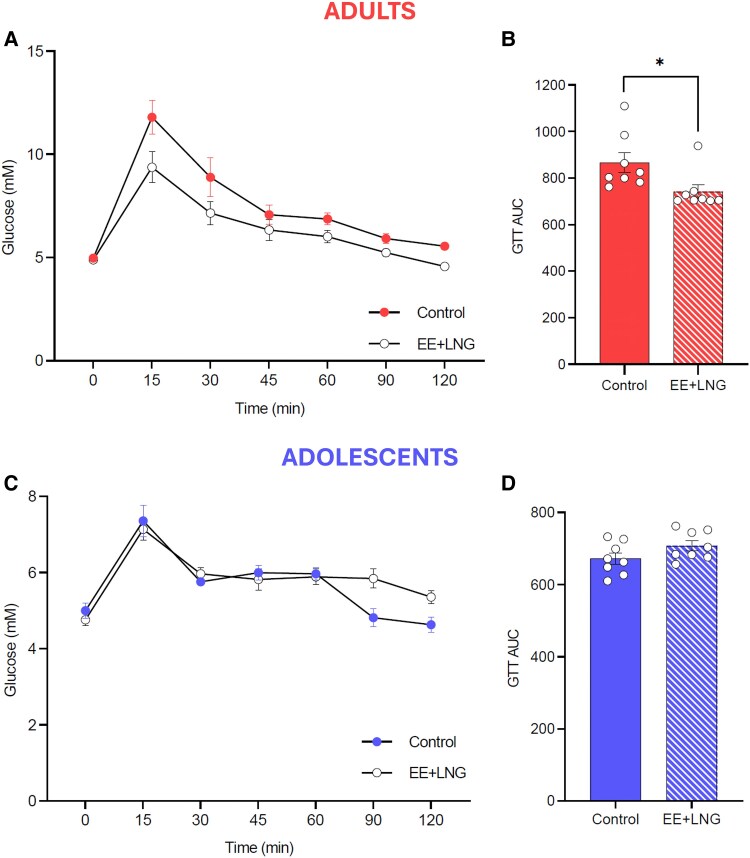
EE + LNG improves glucose tolerance in adults but not adolescents. (A-B) Adults and (C-D) adolescents. (A, C) Blood glucose concentrations (mM) during a 120-min intraperitoneal glucose tolerance test (2 g·kg^−1^), plotted over time (min); symbols represent mean ± SEM. (B, D) Glucose area under the curve (GTT AUC; arbitrary units as plotted), shown as individual animals (points) with group mean ± SEM (bars); vehicle groups are shown with solid bars/symbols and EE + LNG groups with hatched/open bars/symbols. Within each age group, GTT AUC was analyzed using two-tailed independent-samples *t*-tests comparing EE + LNG vs vehicle. Asterisks denote statistically significant treatment differences for the bracketed comparison (*P < .05). Adults: *n* = 8 per group. Adolescents: *n* = 8 per group.

*Adults*: Relative to vehicle-treated rats, EE + LNG-treated adult rats had lower serum levels of C-peptide (*t*_14_ = −2.68, *P* = .018, *d* = −1.3) and leptin (*t*_14_ = −3.15, *P* = .007, *d* = −1.6). Serum concentrations of GLP-1 (*P* = .056), insulin (*P* = .166), glucagon (*P* = .502), ghrelin (*P* = .079), and PYY (*P* = .636) did not significantly differ between adult treatment groups. EE + LNG-treated adult rats had a lower GTT area under the curve compared with vehicle-treated controls (*t*_14_ = −2.43, *P* = .029, *d* = −1.2; Fig. 9A and 9B; Table 5).

**Table 5:** Summary of results from metabolic endpoints from blood sample*s*

	Adults				Adolescents			
Variable	Control	EE + LNG	*P*	*d*	Control	EE + LNG	*P*	*d*
**Metabolic hormone panel**								
GLP-1_(active)_ (pM)	29.89 ± 3.25	22.56 ± 1.33	.056	−1.04	28.29 ± 4.67	14.94 ± 2.62	.023*	1.25
C-peptide (pg/mL)	6232.55 ± 573.85	4222.21 ± 481.71	.009**	−2.68	7678.61 ± 995.29	4691.14 ± 1060.88	.063	1.03
Insulin (μLU/mL)	419.81 ± 66.41	277.56 ± 77.17	.166	−0.73	343.76 ± 45.96	196.98 ± 55.37	.066	1.02
Glucagon (pM)	151.03 ± 24.92	127.06 ± 24.29	.502	−0.34	121.61 ± 21.56	66.13 ± 12.36	.036*	1.12
Ghrelin_(active)_ (pg/mL)	333.01 ± 36.62	234.27 ± 37.09	.079	−0.95	239.33 ± 38.09	250.03 ± 42.04	.855	−0.09
Leptin (pg/mL)	24429.26 ± 5921.53	5463.67 ± 1054.09	.007**	−1.58	9663.20 ± 2023.00	5070.56 ± 1253.50	.069	0.96
PYY (pg/mL)	413.49 ± 58.24	371.90 ± 63.37	.636	−0.24	669.66 ± 60.63	367.87 ± 77.40	.010*	1.53
**Glucose tolerance test (GTT)**								
Glucose AUC (mmol-min/L)	866.02 ± 42.50	741.56 ± 28.56	.029*	−1.22	672.09 ± 15.73	707.48 ± 14.04	.115	−0.84

Circulating metabolic endpoints in EE + LNG-treated and vehicle-treated rats. Mean ± SEM values are presented for adults and adolescents (*n* = 8 per group). The metabolic hormone panel includes GLP-1 (active), C-peptide, insulin, glucagon, ghrelin (active), leptin, and PYY; the glucose-tolerance test (GTT) is summarized as glucose AUC. Group differences were evaluated using post hoc comparisons, and bold denote significant differences (*P < .05; **P < .01). Effect sizes are reported as Cohen's *d*.

*Adolescents*: Unlike adult rats, relative to vehicle-treated rats, EE + LNG-treated adolescent rats exhibited lower serum levels of GLP-1 (*t*_13_ = −2.58, *P* = .023, *d* = −1.4), glucagon (*t*_13_ = −2.34, *P* = .036, *d* = −1.2) and PYY (*t*_13_ = −3.00, *P* = .01, *d* = −1.5). However, serum concentrations of C-peptide (*P* = .063), insulin (*P* = .066), ghrelin (*P* = .855), and leptin (*P* = .069), did not significantly differ between treatment groups in adolescence. There was no significant difference in glucose area under the curve between treatment groups in adolescence (*P* = .115; Fig. 9C and 9D; Table 5).

### Hypothalamic feeding-related gene expression was not significantly altered by EE + LNG in adults or adolescents

*Adults*: Gene-by-gene analyses of hypothalamic ΔCq values did not detect significant treatment differences for AgRP (*t*_14_ = 1.73, *P* = .106, *d* = 0.87), NPY (*t*_14_ = 0.53, *P* = .602, *d* = 0.27), POMC (*t*_14_ = 0.92, *P* = .372, *d* = 0.46), or CART (*t*_14_ = −0.64, *P* = .531, *d* = −0.32). Nevertheless, fold-change estimates relative to vehicle suggested higher expression in EE + LNG-treated adults for AgRP (4.57×), POMC (2.18×), and NPY (1.19×), whereas CART expression was modestly lower (0.87×) (Table 6).

**Table 6:** Summary of hypothalamic feeding-related gene expression (ΔCq) by age and treatment

	Adults				Adolescents			
Gene (ΔCq)	Control	EE + LNG	*P*	Fold change	Control	EE + LNG	*P*	Fold change
Agouti-related peptide (AgRP)	5.495 ± 0.770	3.302 ± 1.007	.106	4.57×	3.553 ± 0.823	3.298 ± 0.689	.816	1.19×
Neuropeptide Y (NPY)	0.884 ± 0.416	0.640 ± 0.528	.602	1.18×	0.530 ± 0.315	0.621 ± 0.499	.811	0.94×
Pro-opiomelanocortin (POMC)	1.385 ± 0.810	0.261 ± 0.859	.372	2.18×	−0.141 ± 0.483	0.284 ± 0.700	.579	0.75×
Cocaine- and amphetamine-regulated transcript (CART)	−0.037 ± 0.336	0.163 ± 0.424	.531	0.87×	−0.227 ± 0.215	0.278 ± 0.378	.148	0.70×

Quantitative PCR was performed on arcuate nucleus punches to quantify AgRP, NPY, POMC, and CART expression. Values are reported as ΔCq (mean ± SEM), where ΔCq was calculated as Cq(target) − Cq using the geometric mean of the reference genes Rpl13a and Tbp. Group differences were evaluated using post hoc comparisons. Fold-change values represent EE + LNG relative to vehicle within each age group and were calculated from group means using the ΔΔCq method (vehicle as calibrator; fold-change = 2^−ΔΔCq). Lower ΔCq values indicate higher relative expression.

*Adolescents*: Similar to adults, no significant treatment differences were detected for AgRP (*t*_14_ = 0.24, *P* = .816, *d* = 0.12), NPY (*t*_14_ = −0.24, *P* = .811, *d* = −0.12), POMC (*t*_14_ = −0.57, *P* = .579, *d* = −0.28), or CART (*t*_14_ = −1.53, *P* = .148, *d* = −0.77). Fold-change estimates relative to vehicle were comparatively small and nonuniform, with AgRP (1.19×), NPY (0.94×), POMC (0.75×), and CART (0.71×) in EE + LNG-treated adolescents (Table 6).

## Discussion

The present research provides insights as to the basis of the reduction in gain of body mass after combined EE + LNG treatment in both adolescence and adulthood. Here we report that chronic exposure to low-dose combined EE + LNG produced a consistent metabolic phenotype in both adults and adolescents: reduced body mass accrual, lower fat mass, preservation of lean mass percentage, reduced food intake, and higher energy expenditure with increased VO_2_ and RER. In addition, the gonadal (gWAT) and inguinal (iWAT) adipose tissue depots were smaller in EE + LNG-treated rats in both age groups. However, age-specific effects emerged in select tissues and endocrine readouts: uterine horn weight increased, and BAT mass decreased with EE + LNG treatment only in adults, glucose tolerance improved only in adults, and the hormone panels diverged, with adults showing lower C-peptide and leptin levels, whereas adolescents showing lower GLP-1, glucagon, and PYY levels. Thus, in the present study, we found both shared mechanisms that generalize across age groups and distinct age-dependent pathways of EE + LNG's effects.

### Validation of female rat model of hormonal contraceptives

Our findings provide validation that the EE + LNG dosing regimen produced endocrine and reproductive outcomes consistent with hormonal contraceptive exposure in humans. In both age cohorts, EE + LNG markedly disrupted normal estrous cyclicity, with vaginal cytology demonstrating a persistent diestrus-like profile across the tracking window, consistent with previous reports (9, 10, 43, 44). Concordantly, circulating LH was reduced in EE + LNG-treated rats in both age groups, and E2 showed the same directional pattern; although the reduction in adolescent E2 did not reach statistical significance, its magnitude was similar to that observed in adults. Importantly, LH and E2 concentrations were lowered but not fully abolished, aligning with clinical observations that HCs attenuate, rather than uniformly eliminate, endogenous gonadotropin and ovarian steroid activity (45-48). At the tissue level, ovarian mass was reduced in both adolescents and adults, consistent with diminished gonadotropin-driven ovarian stimulation (49). In contrast, uterine horn weight increased only in the adult-treated group, reflecting a classic estrogen-responsive trophic effect that has been observed with EE administered alone and in combination with LNG (11, 17, 44, 50). Collectively, these convergent endocrine, cyclicity, and reproductive tissue outcomes verify that our model produces physiologic effects aligned with the contraceptive literature and support its translational relevance.

### EE + LNG reduced body mass regardless of age of exposure

In the present study, EE + LNG reduced body mass in both age cohorts. These findings are more consistent with EE compared with when LNG is administered alone. Ethinyl estradiol administered alone consistently reduced weight gain in adult rats (8, 9, 11, 14-16, 51). We have previously shown that LNG administered alone at 20 µg/kg increases weight gain in adult female rats (9). In addition, unpublished data from our laboratory indicate that LNG administered alone at the same dose similarly increases weight gain in adolescent females relative to vehicle-treated controls (52). Together, these findings indicate that LNG at 20 µg/kg is biologically active in our model. However, because LNG alone promotes weight gain, whereas the EE + LNG combination in the present study markedly reduced food intake and attenuated weight gain, the metabolic phenotype observed here is likely driven by the estrogenic actions of EE, with any LNG-related effects likely counterbalanced or overridden in the combined regimen. We acknowledge that the present design did not include single-hormone arms; therefore, we cannot fully disentangle the independent and interactive contributions of EE and LNG.

In humans, the highest-quality evidence does not support a clinically meaningful effect on body weight in most users (1-4). A meta-analysis found that combined oral contraceptives do not significantly alter body weight or body composition relative to placebo or nonhormonal comparators (2). In population terms, weight trajectories under HC use are well characterized by a near-normal distribution, with most women maintaining weight and smaller proportions gaining or losing, rather than a systematic shift toward gain (3, 4). This distributional pattern mirrors the body mass findings in our adult cohort: relative to controls, HC-treated rats showed a distribution centered near 0% change with a slight trend toward modest weight loss; most animals clustered close to no change, and the remainder split between small losses and small gains. Nevertheless, our 16-day regimen models the early phase of exposure and captures acute metabolic responses to EE + LNG. Since human use typically extends over months to years, future work should test longer dosing intervals to assess durability and dose-duration dependencies of these effects

### EE + LNG increased energy expenditure, regardless of age of exposure

In the present study, reduced caloric intake alone does not fully explain the diminished gain in body mass we show with EE + LNG in both age cohorts, because EE + LNG also resulted in a higher energy expenditure. In both the adult and adolescent cohorts, total VO_2_ consumption and daily energy expenditure were higher in the EE + LNG groups. This elevation was paired with a slightly higher respiratory exchange ratio indicating a modest shift toward carbohydrate oxidation (53), whereas cage ambulation or locomotion was unchanged.

The combined pattern of glucose handling, adipose remodeling, and UCP1 abundance suggests that EE + LNG increases whole-body metabolic rate in both age cohorts, but the tissues most closely linked to this effect and the downstream metabolic consequences differ by developmental stage. In adults, the increase in energy expenditure coincided with improved glucose tolerance, as reflected by a lower GTT AUC. This profile is consistent with enhanced peripheral glucose disposal during sustained metabolic activation. Brown and beige adipocytes can act as major glucose sinks during thermogenic activation (54, 55), and the adult phenotype of reduced gonadal and inguinal white adipose depots together with reduced BAT mass may reflect adipose remodeling under chronic EE + LNG exposure. However, UCP1 protein content in BAT and gWAT did not differ by treatment in adults. This lack of a UCP1 response in adults may reflect age-related constraints on white adipose plasticity, as beiging capacity generally declines with maturation (56-58), potentially rendering adult WAT less responsive to EE + LNG-induced induction of UCP1 content. These findings indicate that the elevated energy expenditure and glycemic benefit are not readily explained by higher endpoint UCP1 abundance in these depots. A plausible interpretation is that EE + LNG may increase thermogenic activity without increasing steady-state UCP1 protein levels, or that energy dissipation occurs through pathways (ie, muscle nonshivering thermogenesis) not captured by UCP1 content alone.

In adolescents, EE + LNG similarly increased VO_2_ and total energy expenditure, yet did not improve glucose tolerance. This dissociation suggests that elevated metabolic rate does not necessarily translate into measurable glycemic benefit early in development. The adolescent adipose phenotype also differed from adults. Brown adipose tissue mass was unchanged, while inguinal and gonadal white adipose depots were markedly reduced. In addition, adolescents showed higher UCP1 content in gWAT under EE + LNG compared with vehicle, consistent with increased recruitment of beige-like characteristics within a white adipose depot. This increase in gWAT UCP1 in adolescents is consistent with the greater beiging capacity of younger white adipose tissue, which is generally more plastic and more responsive to stimuli that promote UCP1 induction (59, 60). Although BAT UCP1 content was unchanged, the increase in gWAT UCP1 observed selectively in adolescents provides evidence for enhanced thermogenic capacity within white adipose tissue and may contribute to the larger magnitude of the EE + LNG-associated increase in energy expenditure in this age group. The absence of a corresponding reduction in GTT AUC suggests that developmental factors modulate how adipose remodeling and metabolic activation influence glucose homeostasis.

Overall, EE + LNG increased energy expenditure in both ages, but the accompanying metabolic signatures were distinct. In adolescents, increased energy expenditure occurred alongside reduced white adipose depot mass and higher gWAT UCP1, consistent with beiging, without measurable improvement in glucose tolerance. In adults, increased energy expenditure was accompanied by improved glucose tolerance and reductions in both white adipose and BAT mass, but without detectable changes in UCP1 content in BAT or gWAT at endpoint. Together, these findings suggest that UCP1 abundance at endpoint is not the sole determinant of EE + LNG-related metabolic activation and highlight developmental stage as an important moderator of adipose remodeling and glycemic outcomes.

There is growing evidence that estrogen-containing contraceptives modestly raise resting energy expenditure (REE) or basal metabolic rate (BMR) in humans (61). A recent systematic review concluded that HCs are associated with elevated REE, with increases up to approximately +208 kcal per day vs baseline or nonuser conditions, and proposed estrogen-mediated enhancements of thermogenesis and mitochondrial activity as plausible mechanisms (61). Although not all human studies observe a BMR increase in HC users (62), the balance of evidence favors a small upward shift in energy expenditure (61). This interpretation is consistent with our observation of elevated REE in EE + LNG-treated rats.

### EE + LNG reduced fat mass and maintained lean mass

The combined effects of reduced caloric intake and increased energy expenditure had marked effects on body composition as measured by DXA and adipose tissue depots. In both age cohorts, EE + LNG prevented the fat-mass gain observed in vehicle controls and maintained lean-mass percentage at the study endpoint. We did, however, observe a bimodal pattern of results in the adolescent cohort on fat mass percentage. Visual inspection of the raw DXA outputs indicated that the bimodal distribution of adolescent fat mass percentage may have reflected a cohort-related shift rather than treatment heterogeneity. Accordingly, we re-analyzed the adolescent endpoint using a model that included *cohort* as an additional factor; this sensitivity analysis did not alter the primary conclusion, as vehicle-treated adolescents still exhibited significantly higher final DXA-derived fat mass percentage than EE + LNG-treated adolescents. Notably, this cohort-related pattern appeared specific to the DXA fat mass percentage measure, as we examined all other study endpoints and did not observe cohort effects. The changes in body composition observed here run counter to those reported by Porwal et al (2023), who administered a lower dose and for a longer time period than the present study to 6-8 week-old Sprague-Dawley rats, and found diminished lean mass and elevated fat mass compared with controls. However, this effect was no longer observed after 7 months on HCs, suggesting the effects of EE + LNG on body mass may be transient (12). Differences in body composition results between this study and that of Porwal et al (2023) may therefore be due to differences in dosing, length of HC exposure, and rat strain.

### Food intake was reduced in both age groups, but hypothalamic gene expression and metabolic hormone responses were age-specific

The robust reduction in cumulative food intake observed in both adolescents and adults indicates a potent anorexigenic effect of EE + LNG exposure. Notably, this hypophagic phenotype occurred in the context of lower circulating leptin in both age cohorts. Because leptin typically suppresses appetite through inhibition of AgRP/NPY neurons and activation of POMC pathways, reduced leptin would ordinarily be expected to *increase* orexigenic drive (63-65). The presence of marked hypophagia despite reduced leptin therefore suggests that EE + LNG imposes a satiety constraint that overrides canonical adiposity feedback, and that the endocrine adaptations observed at endpoint likely reflect homeostatic counter-regulation rather than the proximal cause of reduced intake.

EE + LNG did not significantly alter hypothalamic feeding-related gene expression. Nonetheless, the descriptive fold-change estimates may still be informative as exploratory indices of the direction and relative magnitude of expression differences. In adults, the largest fold-change estimate was observed for AgRP, with a more modest increase in POMC, whereas changes in NPY and CART were comparatively small. In adolescents, fold-change estimates were generally smaller and less differentiated across genes. Because these fold-change values were not supported by statistically significant gene-specific effects, they should be regarded as hypothesis-generating rather than confirmatory.

The adult pattern is consistent with the possibility of a compensatory response to sustained negative energy balance. Specifically, the numerically larger fold-change estimate for AgRP in adults may be compatible with a homeostatic attempt to restore food intake in the face of reduced adiposity and lower leptin (66, 67). However, this interpretation remains speculative and cannot be taken as evidence of a treatment-induced increase in AgRP expression. Similarly, the smaller and more diffuse fold-change pattern in adolescents may suggest that hypothalamic transcriptional adaptation to EE + LNG is less pronounced or differently timed during adolescence, but again, this cannot be concluded definitively from the present data.

Overall, the combination of reduced food intake, lower leptin, and the absence of statistically significant changes in hypothalamic gene expression suggests that the hypophagic effects of EE + LNG are unlikely to be explained by robust endpoint differences in AgRP, NPY, POMC, or CART mRNA expression. Instead, any contribution of hypothalamic feeding circuits may be subtle, temporally dynamic, or expressed at the level of neural activity or peptide release rather than endpoint transcript abundance (68-70).

We also observed an increase in water consumption among all EE + LNG-treated rats, although this was significant only in the adult cohort. Increased water intake is a predictable consequence of higher energy turnover and thermogenesis (71, 72). This finding may seem at odds with the well-known hypodipsic effect of endogenous E2 during normal feeding (73), however, E2 stimulates water intake when food is withheld (30). Given the marked reduction in food intake in EE + LNG-treated animals in the present study, concomitant exposure to the potent estrogen EE may have increased thirst, consistent with the known E2 stimulation of water intake during food restriction (30). The rise in water intake could therefore represent a compensatory response to reduced dietary intake. Another possible explanation for the increased water intake is that LNG has partial mineralocorticoid-receptor activity (74) which can enhance renal sodium retention (75) and can indirectly promote thirst (76, 77).

EE + LNG produced a distinct endocrine signature in adult rats that dovetails with the observed changes in body mass. Serum C-peptide concentrations were lower in the EE + LNG groups in adulthood. In this context, the lower GTT AUC alongside reduced insulin and C-peptide indicates more efficient glucose disposal for a given insulin signal in adults (78). Adipose tissue could also contribute to faster glucose clearance if thermogenic pathways are engaged, because activated brown and beige adipocytes avidly take up glucose during thermogenesis. Fasting leptin also declined in EE + LNG-treated adults, aligning with the marked loss of adipose tissue, and consistent with the notion that leptin tracks with fat-mass rather than directly driving food intake under these conditions (79). However, in adulthood, GLP-1, PYY, and ghrelin were unaltered, suggesting that peripheral satiety hormones did not mediate the hypophagia.

In adolescents, the hormonal pattern diverged from that of the adult cohort and did not map neatly onto the hypophagia and weight attenuation observed. EE + LNG lowered GLP-1, glucagon, and PYY, yet left leptin, insulin, and C-peptide unchanged. Because GLP-1 and PYY are classically anorexigenic (80, 81), their reduction would be expected to increase food intake, making it unlikely that gut-derived peptides explain the decrease in consumption. Likewise, lower glucagon might be expected to blunt hepatic glucose output (82, 83), but in the adolescent cohort, GTT AUC was not different from controls. The discrepant endocrine profiles between adolescent and adult rats likely reflect fundamental differences in developmental stage and the way synthetic steroids interact with the maturing HPG and metabolic axes. In adults, the coordinated reduction in C-peptide, insulin, and adiposity alongside improved glucose handling suggests that EE + LNG engages mature feedback circuits, enhancing insulin sensitivity and favorably remodeling energy balance. By contrast, adolescence is characterized by rapidly shifting hormone milieus (84, 85), higher metabolic demand (31, 32, 86), dynamic insulin sensitivity (87), and ongoing maturation of central appetite and glucose-regulating networks (88). Synthetic steroids introduced during this period may interact unpredictably with these immature systems. Given that the reduction in food intake did not appear to be related to robust changes in hypothalamic orexigenic transcriptional responses or consistent alterations in appetite-related hormones the hypophagia evident in both age cohorts may also reflect a broader reduction in food reward sensitivity or palatability. Indeed, EE + LNG co-administration diminished voluntary sucrose intake in the sucrose-preference test in mice (89).

Nevertheless, the integrated profile of reduced food intake, heightened energy expenditure, and decreased adiposity, remains congruent with classic estrogen receptor (ER)-α activation in hypothalamic and peripheral tissues (61, 90). Ethinyl estradiol is a synthetic estrogen with roughly threefold higher affinity for estrogen receptor (ER) α than E2 and lower, but measurable, activity at ERβ (91). Levonorgestrel, a 19-nortestosterone progestin, binds the progesterone receptor with high affinity, also engages the androgen receptor, and shows lower binding to mineralocorticoid receptors, yielding partial androgenic activity and different steroid-receptor crosstalk than progesterone (74, 92, 93). Accordingly, phenotypes in rats treated with EE + LNG vs vehicle likely reflect these distinct receptor affinities, isoform selectivity, and downstream signaling relative to endogenous E2, progesterone, and testosterone.

The metabolic phenotype elicited by EE + LNG, that is, reduced food intake, elevated energy expenditure, and a carbohydrate-skewed fuel profile as measured by respiratory exchange ratio, has ramifications that extend beyond somatic outcomes. The brain is an energetically demanding organ (94) and shifts in systemic substrate availability and resting metabolic rate can alter cerebral glucose utilization (95), synaptic plasticity (96), and ultimately performance on cognitive tasks (97). Experiments that involve food reward or rely on motivated feeding behavior may be confounded if hormone-treated animals voluntarily consume food to a lesser extent than controls. Finally, when experiments impose caloric restriction, a higher basal energy cost in EE + LNG rats means that each gram of chow yields fewer net calories, potentially exaggerating “restriction” relative to controls. In sum, the consummatory and metabolic changes documented here underscore the need to integrate whole-body physiological endpoints with behavioral readouts when dissecting the neurobiological consequences of HCs.

### Conclusions and future directions

Combined EE + LNG treatment elicited a robust metabolic phenotype in female rats, characterized by reduced food intake, diminished body mass accrual, lower adiposity, preserved lean mass percentage, and elevated energy expenditure in both adolescence and adulthood. Yet, despite these broadly similar phenotypic outcomes, the physiological correlates associated with this response were age specific. Adults showed endocrine changes suggestive of improved glucose handling together with reduced BAT mass and lower white adipose depots, whereas adolescents displayed a distinct metabolic hormone profile and increased gWAT UCP1 content consistent with greater thermogenic plasticity. Thus, developmental age shaped not whether EE + LNG altered metabolism, but how those effects were manifested across tissues and endocrine systems. These findings demonstrate that similar outward metabolic phenotypes can arise from different underlying physiological adaptations depending on developmental stage. A limitation of the present study is that, although commercially available rat-specific ELISA kits were used according to the manufacturer's instructions and assay performance was monitored by duplicate measurement and intraassay variability, we did not perform a full independent in-house validation of assay accuracy under our specific experimental conditions. Future work should extend treatment duration, further dissect estrogenic vs progestin-specific actions, and more directly characterize the mechanisms linking contraceptive hormone exposure to adipose remodeling and energy expenditure. Together, these findings contribute to a more nuanced understanding of how low-dose contraceptive hormones modulate energy balance across developmental stages.

## Data Availability

Some or all datasets generated during and/or analyzed during the current study are not publicly available but are available from the corresponding author on reasonable request.
